# Nitric Oxide in the Offensive Strategy of Fungal and Oomycete Plant Pathogens

**DOI:** 10.3389/fpls.2016.00252

**Published:** 2016-03-04

**Authors:** Magdalena Arasimowicz-Jelonek, Jolanta Floryszak-Wieczorek

**Affiliations:** ^1^Department of Plant Ecophysiology, Faculty of Biology, The Adam Mickiewicz UniversityPoznan, Poland; ^2^Department of Plant Physiology, The University of Life Sciences in PoznanPoznan, Poland

**Keywords:** pathogen-derived nitric oxide, nitrosative stress resistance, defense response, biotrophic pathogens, necrotrophic pathogens

## Abstract

In the course of evolutionary changes pathogens have developed many invasion strategies, to which the host organisms responded with a broad range of defense reactions involving endogenous signaling molecules, such as nitric oxide (NO). There is evidence that pathogenic microorganisms, including two most important groups of eukaryotic plant pathogens, also acquired the ability to synthesize NO *via* non-unequivocally defined oxidative and/or reductive routes. Although the both kingdoms *Chromista* and Fungi are remarkably diverse, the experimental data clearly indicate that pathogen-derived NO is an important regulatory molecule controlling not only developmental processes, but also pathogen virulence and its survival in the host. An active control of mitigation or aggravation of nitrosative stress within host cells seems to be a key determinant for the successful invasion of plant pathogens representing different lifestyles and an effective mode of dispersion in various environmental niches.

## Introduction

During plant–pathogen interactions a decisive role is played by the rate and intensity of reactions induced by both adversaries. In the initiation, coordination and transfer of information on the appearing threat a particularly significant role is exerted by endogenous signaling molecules, such as nitric oxide (NO). This free radical gas can diffuse rapidly through biological membranes and it is capable of acting as a transient, local, intra-, and intercellular signal within species from every biological system.

The signaling function of NO has been shown during both highly conserved pathogen-associated molecular pattern (PAMP) triggered immunity (PTI) and in a highly specific effector-triggered immunity (ETI), often accompanied by the hypersensitive response (HR) at the site of attempted host colonization (e.g., Mur et al., 2006; Floryszak-Wieczorek et al., 2007; Asai and Yoshioka, 2009; Schlicht and Kombrink, 2013). Importantly, the generation and potential functions of NO during these interactions have so far been analyzed solely from the point of view of the host plant. Many years ago Mur et al. (2006) highlighted that the role of NO within the pathogen was too often ignored when considering plant-pathogen interactions.

The plant–pathogen system is dynamic and in the course of evolutionary changes pathogens have developed numerous invasion strategies, to which the host organism has responded with an extensive range of defense traits known as “fight for their lives”. It is known from the scarce studies that also certain pathogenic microorganisms are capable of synthesizing NO, although the role of NO in this systematically heterogeneous group of organisms has not been specified. Definitely the greatest amount of experimental data, both in terms of the sources of synthesis and the potential function of NO in the host-pathogen interactions, has been supplied by studies on model bacterial and fungal human pathogens.

Since pathogens may use NO to its own benefit and concurrently, NO may prime the host organisms and activate their defense, this review focuses on the mode of NO action in two most important groups of eukaryotic plant pathogens, i.e., fungi and oomycetes.

## The Origin of NO in Fungal and Oomycete Plant Pathogens

Pathogens are able to produce NO; however, the origin of NO seems to be as unclear as in plants (Arasimowicz-Jelonek and Floryszak-Wieczorek, 2014). In general, the biosynthetic pathways of NO in fungal and oomycete phytopathogens can be classified as either oxidative or reductive in operation.

The oxidative route involves NOS-like activity, which was evidenced in various groups of fungi. A pharmacological approach using mammalian NOS inhibitors, i.e., L-NAME and/or 1-[2-(Trifluoromethyl)phenyl]imidazole (TRIM), revealed a limited NO production including phytopathogenic fungi *Macrophomina phaseolina, Blumeria graminis*, and *Colletotrichum coccodes*, the mycoparasitic fungus *Coniothyrium minitans* and the aquatic fungus *Blastocladiella emersonii* (Wang and Higgins, 2005; Prats et al., 2008; Vieira et al., 2009; Li et al., 2010; Sarkar et al., 2014). Moreover, the NOS-like activity has been confirmed by measuring citrulline formation from ^3^H-labeled arginine in the endophytic and pathogenic fungus *Neurospora crassa*, the non-pathogenic Mucorales *Phycomyces blakesleeanus* and *C. minitans* (Ninnemann and Maier, 1996; Maier et al., 2001; Li et al., 2010). Importantly, some representatives of ascomycetes and zygomycetes possess tetrahydrobiopterin, a typical mammalian NOS cofactor essential for NO synthesis (Maier and Ninnemann, 1995). Additional experiments evidenced that the catalytic activity of the fungal NOS-like enzyme depends on NADPH and involves calcium ions (Vieira et al., 2009).

Based on genome analysis NOS-like sequences were found in genomes of fungal species from the genus *Aspergillus* (*A. flavus*, *A. oryzae*, and *A. niger*) as well as *Glomerella graminicola*, the teleomorph stage of the anthracnose pathogen *Colletotrichum graminicola* (Turrion-Gomez and Benito, 2011; Sarkar et al., 2014). More recently, NOS-like protein with conserved amino acid sequences was found in the genome of *M. phaseolina*, a necrotrophic fungus causing charcoal rot disease (Sarkar et al., 2014). What is more, multiple alignments of NOS sequences followed by motif enrichment analysis have generated two motifs, one in the oxygenase domain and the other in the flavodoxin/NO synthase domain presented in separate open reading frames (ORFs). As indicated by Sarkar et al. (2014), these motifs are conserved among the five necrotrophic plant pathogens including *M. phaseolina*, *Neofusicoccum parvum*, *Colletotrichum gloeosporioides*, *G. graminicola*, and *A. oryzae*.

Available data indicate that NOS-like activity in fungi could be greatly varied. For example, in mycelia of *P. blakesleeanus* it is 10 pmol/mg/min (Ninnemann and Maier, 1996) and in fruiting bodies of white-rot basidiomycete *Flammulina velutipes* it amounts to 500 pmol/mg/min (Song et al., 2000). In *C. minitans* the highest level of NOS-like activity, amounting to 20 pmol/min/mg, was recorded during conidiation (Li et al., 2010), suggesting that this route of NO synthesis is closely regulated depending on the species, developmental stage and environmental conditions.

Evidence for the existence of an nitrate redctase (NR) dependent pathway of NO biosynthesis in *A. nidulans* was recently provided by Marcos et al. (2016). Although this reductive route was found to be functional during growth in both liquid and solid media, NR-dependent NO production was more abundant on solid media, where reproductive development occurred.

Fungi are able to produce NO as a result of denitrification processes, catalyzed by three enzymes: nitrate reductase (Nar), nitrite reductase (Nir), and NO reductase (Nor) (Ye et al., 1994; Morozkina and Kurakov, 2007). The denitrifying system coupled with the mitochondrial electron transport chain facilitates anaerobic respiration associated with ATP synthesis under hypoxia conditions. Nitrite reductases located in the intermembrane space of the fungal mitochondria have been shown to reduce NO_2_^-^ to NO in a NADP-dependent manner (Röszer, 2012). A dissimilatory nitrite reductase was found in phytopathogens *Fusarium oxysporum* and *Cylindrocarpon tonkinense* as well as in a potentially pathogenic for humans yeast *Fellomyces fuzhouensis* (Abraham et al., 1993; Kobayashi and Shoun, 1995; Kobayashi et al., 1996; Uchimura et al., 2002). Moreover, using combination of the membrane inlet mass spectrometry (MIMS) and the restriction capillary inlet mass spectrometry (RIMS) techniques a nitrite-induced NO production has been demonstrated from cultures of plant pathogenic fungi *Botrytis* and *Fusarium* sp. and the oomycete *Pythium* (Conrath et al., 2004)

Interestingly, NO synthesis in the rice blast fungus *Magnaporthe oryzae* was associated neither with nitrite-dependent nor with the arginine-dependent pathway (Samalova et al., 2013). By creating mutants with a single and double knockout of genes potentially involved in NO synthesis, including NOS-like genes (*NOL2* and *NOL3*), nitrate (*NIA1*) and nitrite reductase (*NII1)*, the authors revealed that NO is not generated by the candidate proteins. A mammalian NOS inhibitor, L-NAME applied to a necrotroph *Botrytis cinerea* growing on a medium and *in planta* did not limit NO production either (Turrion-Gomez and Benito, 2011). The pathogen was also incubated with NO_3_^-^ to identify the source of NO production *via* nitrate reductase, but NO signal was not detected implying that a physiological and genetic system other than NOS and NR is responsible for NO production in this plant pathogen (Turrion-Gomez and Benito, 2011). A plausible explanation for this elusive route of NO synthesis could involve an unidentified complex system of a fungus-type enzyme catalyzing NO production.

As suggested by Röszer (2012), the oxidative route of NO formation might be dominant in pathogen units under aerobic conditions and localized in the cytoplasm, whereas enzymes responsible for NO production under hypoxia or anoxia could involve mitochondrial cytochrome-c oxidase and mitochondrial nitrite reductase.

## NO as a Developmental Signal in Fungal and Oomycete Pathogens

Recent studies have indicated that NO may play an important role in signaling networks in fungi. Based on the pharmacological approach (including also non-pathogenic fungi) it was shown that NO participates in the development of various fungal structures including sporangiophores, conidia, cleistothecia, pycnidia and appresoria (Maier et al., 2001; Wang and Higgins, 2005; Gong et al., 2007; Prats et al., 2008; Baidya et al., 2011). However, the intensity of NO generation and its location is strictly dependent on the developmental stage of the pathogen. Bio-imaging with fluorochrome DAF-FM DA showed NO presence in *C. coccodes* conidia, germ tubes, and immature appressoria, but the strongest NO-dependent fluorescence was observed in the cells with reduced cytoplasm in the conidium with a mature appressorium (Wang and Higgins, 2005). Also in the *B. cinerea* system the production of NO was detected in all fungal developmental stages, starting from spores up to mature mycelium (van Baarlen et al., 2004; Turrion-Gomez and Benito, 2011). A strong NO signal was found in young pycnidia of *C. minitans* and in the undifferentiated tissue of pycnidial primordia; in turn, weak fluorescence signals were observed in growing hyphal tips or hyphae, where pycnidia or primordia did not develop (Gong et al., 2007).

In general, both sexual and asexual reproduction in fungi was documented to be dependent on NO generation. What is more, NO could control a threshold to switch developmental phases. It was found that a NO-releasing compound (DETA NONOate) reduced asexual development in *A. nidulans* which has a limited, but significant, phytopathogenic potential (Dean and Timberlake, 1989). In turn, formation of sexual structures was increased after NO supplementation in several fungal species, including species from the genera *Aspergillus* and *Neurospora*, as well as the species *F. velutipes* (Song et al., 2000; Baidya et al., 2011). Alterations in *A. nidulans* conidiation induced by exogenous NO may be due to NO impairing the transcriptional activation of structural sporulation specific genes (Chiuchetta and Castro-Prado, 2005). NO levels influenced the balance between conidiation and sexual reproduction, since an artificial strong elevation of NO levels reduced conidiation and induced the formation of cleistothecia. As it was found by Marcos et al. (2016), different NO levels affected the expression of the regulator of sexual development *nsdD* and the regulator of conidiation *brlA*. Baidya et al. (2011) also showed that NO is involved in the switch of developmental phases. Deletion of *fhbA*, a gene encoding flavohaemoglobin (Fhb) protein involved in the reduction and detoxification of NO, resulted in increased Hülle cell production which nurse the young fruiting body during development (Baidya et al., 2011). This result suggests that *fhbA* is likely to suppress sexual development under stressful conditions accompanied by reactive nitrogen species (RNS) overproduction (Dyer and O’Gorman, 2012).

Nitric oxide also controls the development of sporangiophores in the zygomycete *P. blakesleeanus* (Maier et al., 2001). Exposure to light signals activated asexual reproduction concomitant with NO emission within developing cells of the fungus. Exogenous NO was able to mimic the light effect on sporangiophore formation indicating that NO could function as a light sensor molecule during light-mediated sporulation. The photoconidiation process in ascomycete *N. crassa* is also dependent on NO (Ninnemann and Maier, 1996). However, NO donor inhibited light-stimulated conidiation in *N. crassa*, whereas specific inhibitors of NOS activity enhanced conidiation in darkness and in the light. The role of NO in fungal physiology is related to spore germination as well. NO synthesis was found during germination of *C. coccodes* spores. Since NO trapping accelerated germination and exogenous NO delayed this phenomenon the authors suggested that a specific NO threshold could control the time of exit from spore dormancy (Wang and Higgins, 2005).

The sporulation of the blastocladiomycete *B. emersonii* was accompanied not only by an increased level of NO and NO-derived compounds but also by the expression of genes coding for guanylyl cyclase and cGMP phosphodiesterase. Using the pharmacological approach Vieira et al. (2009) demonstrated that the Ca^2+^-NO-cGMP signaling pathway facilitated control of zoospore biogenesis in the aquatic saprophytic fungus. Further support for the signaling role of NO during fungal development has been provided by Gong et al. (2007). The plant pathogen *Cryphonectria parasitica* failed to produce pycnidia in response to both L-NAME and the cGMP blocker (6-anilinoquinoline-5, 8-quinone), suggesting that the NO-mediated signal for conidiation may be common to multiple fungal genera, which produce pycnidia (Gong et al., 2007). Importantly, the dynamics of NO synthesis was closely related to changes in cGMP levels during pycnidial development.

## NO Offensive and Necrotrophic Pathogens

Nitric oxide was found to be produced by various plant necrotrophs, including *B. cinerea*, *A. nidulans, M. phaseolina*, *F. oxysporum*, and *C. coccodes* (Conrath et al., 2004; Wang and Higgins, 2005; Floryszak-Wieczorek et al., 2007; Turrion-Gomez and Benito, 2011; Sarkar et al., 2014). Based on a model necrotroph *B. cinerea* NO generation was detected during both saprophytic growth and *in planta* (Conrath et al., 2004; Floryszak-Wieczorek et al., 2007). Based on DAF2-DA fluorochrome, the presence of NO was found in hyphae and spores of *B. cinerea* growing on a solid medium (Floryszak-Wieczorek et al., 2007). What is noteworthy, the necrotroph contact with pelargonium leaf tissue of the susceptible genotype resulted in the acquisition of the ability to generate much greater amounts of NO, favoring necrotic death of host cells and in consequence – disease development. The NO-dependent fluorescence surrounded germinating spores and mycelium of *B. cinerea* growing both on a complete medium and *in planta*, indicated the diffusion of NO produced inside the fungal cells (Turrion-Gomez and Benito, 2011). The observed NO spreading outside the fungal structures could have important physiological consequences in the establishment and progress of the disease, since pathogen-derived NO could reach plant cells and contribute to the hypersensitive cell death, facilitating subsequent tissue colonization (Turrion-Gomez and Benito, 2011; Arasimowicz-Jelonek and Floryszak-Wieczorek, 2014). Noteworthy, a compound required for full virulence of *B. cinerea*, the endopolygalactouronase 1 (BcPG1) was evidenced to trigger a phosphorylation-dependent NO production in grapevine cells (Vandelle et al., 2006). Thus, NO overaccumulation *in planta* induced by pathogen or originating both from the pathogen and the host plant might accelerate the spread of infection and constitute a significant element determining success of the necrotrophic aggressor. A strong accumulation of NO in host tissue correlated with disease development was also observed in the compatible lily–*Botrytis elliptica* interaction (van Baarlen et al., 2004). In the susceptible jute–*M. phaseolina* interaction NO overproduction followed 20 dpi and coincident with NO-derived compound accumulation localized in the vascular bundle region containing invaded mycelium and micro-sclerotia (Sarkar et al., 2014). It should be noted that the necrotroph-induced NO generation in plant cells could also correlate with enhanced disease resistance. An early and only transient NO burst synchronized with ROS generation was found to positively modulate resistance in various plants under attack by necrotrophic fungal pathogens, such as *Botrytis cinerea* or *Sclerotinia sclerotiorum* (Mur et al., 2006; Floryszak-Wieczorek et al., 2007; Asai and Yoshioka, 2009; Perchepied et al., 2010).

Production of NO could also have implications in virulence of fungal pathogens *via* regulation of mycotoxin biosynthesis. For example, the Fhb gene *fhbA* influences mycotoxin biosynthesis in necrotrophic *A. nidulans* Δ*fhbA* (or Δ*fhbA* Δ*fhbB*), since deletion mutants showed a reduction of sterigmatocystin production (Baidya et al., 2011). The diminished pool of mycotoxins coincided with a decrease in the expression of *aflR*, a transcription factor necessary for the activation of the sterigmatocystin gene cluster. The NO-releasing compound application to Δ*fhbA* strains resulted in an increase of *aflR* expression levels and in the recovery of mycotoxins near the wild-type levels (Baidya et al., 2011).

## NO Offensive and Hemi-/Biotrophic Pathogens

Pathogens with the hemibiotrophic and biotrophic life strategies are able to produce NO. This RNS was found to be generated by mycelia of *Oidium neolycopersici* (Piterková et al., 2009), *B. graminis* f. sp. *hordei* (Prats et al., 2008), *M. oryzae* (Samalova et al., 2013) and by hyphae of the oomycete *Bremia lactucae* (Sedlárová et al., 2011).

In *B. lactucae* the presence of NO was observed in the infection structures of the pathogen grown both on susceptible and resistant lettuce genotypes; however, the plant genotype determined timing of the pathogen development. A strong NO signal was detected in the tip of the germ tube and appressorium, which is a prerequisite for tissue penetration. A weaker NO signal was detected in developing primary and secondary vesicles, intracellular hyphae and in haustoria on susceptible lettuce (Sedlárová et al., 2011). The genotype with an abundant HR showed NO generation frequently localized in penetrated cells undergoing HR before the occurrence of detectable necrosis (Sedlárová et al., 2011).

Rice blast fungus *M. oryzae* produced NO during germination and early development, and was critically required for the progress of appressorium formation. Importantly, the elimination of NO produced by *M. oryzae* significantly reduced the level of infection in a compatible interaction, confirming that the pathogen-derived NO is required for successful host colonization by the hemibiotrophic pathogen (Samalova et al., 2013). Most recently, Zhang et al. (2015) revealed that the *M. oryzae* gene *MoSFA1* coding S-(hydroxymethyl)-glutathione dehydrogenase involved NO metabolism through the reduction of S-nitrosoglutathione (GSNO) contributing to full virulence in the pathogen. *MoSFA1* mutants showed attenuated virulence on rice cultivar CO-39, as well as severe reduction of conidiation and appressorium turgor pressure. Importantly, the virulence of *MoSFA* mutants on wounded rice leaves was not affected, indicating that MoSFA1 significantly contributes to virulence during penetration or the biotrophic phase of the pathogen.

Differentiation of infection structures was also found to be determined by NO in the biotroph *B. graminis* f. sp. *hordei*. NO trapping as well as blocking of NOS-like activity significantly reduced the number of appressorial lobes, which in consequence affected host cell penetration (Prats et al., 2008).

In contrast to plant-necrotroph interactions, tomato powdery mildew development was not accompanied by NO over-accumulation in host cells, although pathogen-derived NO could favor infection. NO signal was detected in *O. neolycopersici* conidia, germ tubes and appressoria developed on various genotypes of *S. lycopersicum* at 24 and 48 hpi. At later stages of pathogenesis NO was absent in spreading structures of the pathogen in susceptible tomato and only attacked cells of moderately resistant and highly resistant plants showing a strictly localized NO generation (Piterková et al., 2011).

Since the functional role of NO in living organisms may be realized directly through the involvement of NO in the post-translational modification, NO is likely to have significant implications in pathogen virulence also via the nitration/S-nitrosylation phenomenon. The analysis of a crude extract of the plant biotrophic fungus *Plasmopara halstedii* revealed the presence of nitrated proteins; however, their role remains unknown (Chaki et al., 2009).

## Pathogen Survival Under NO Stress

Colonization of the host tissues by pathogens often results in an over-production of NO and NO-derived molecules, which create boosted and pathophysiological levels of RNS, defined as nitrosative stress (Arasimowicz-Jelonek and Floryszak-Wieczorek, 2014). Therefore, the nitrosative stress response seems to be notably important during the early stages of infection when the fungus is battling with host defenses.

Although NO over-accumulation could favor necrotroph development and/or host colonization, most of the published data rather support an adverse effect of NO on fungal growth (Schlicht and Kombrink, 2013). It remains controversial whether NO itself can kill pathogens; however, the potential antimicrobial effect of NO on pathogens has been experimentally proven. Gaseous NO inhibited mycelial growth, spore germination and sporulation of three plant pathogenic fungi, including *A. niger*, *Monilinia fructicola*, and *Penicillium italicum* under *in vitro* conditions, indicating a direct effect of exogenous NO on fungal metabolism (Lazar et al., 2008). The inhibitory effect of NO on fungal growth and spore germination of the plant pathogen *P. expansum* was related to increased levels of intracellular ROS and elevated carbonylation damage. Simultaneously, the activities of SOD and CAT as well as ATP content were diminished in response to the NO modulator (Lai et al., 2011). Moreover, the *35S::nNOS Arabidopsis* line, which contained constitutively enhanced NO levels due to the expression of the rat neuronal NOS, rendered the plants more resistant to infection by a biotrophic fungus *Golovinomyces orontii* (Schlicht and Kombrink, 2013). This transgenic *Arabidopsis* line displayed also enhanced resistance to a bacterial pathogen *P. syringae* and enhanced tolerance to salt and drought. Since the enhanced resistance was associated with SA accumulation and SA-responsive defense genes expression, the authors speculated that restriction of fungal growth was the result of diverse NO-mediated plant defense components; however, a direct effect of NO on biotroph development may not be excluded (Schlicht and Kombrink, 2013).

Phytopathogens cope both with innate NO and with plant-derived NO. In response to these huge amounts of NO pathogenic microorganisms have evolved constitutive and inducible mechanisms to prevent the adverse effects of NO, helping them to survive during the contact with host cells (Arasimowicz-Jelonek and Floryszak-Wieczorek, 2014). Thus, the pathogen metabolic equipment to counteract nitrosative stress might be implicated in its virulence. Among inducible mechanisms, the detoxification of NO governed by the evolutionarily ancient Fhb is the best described for model fungi and human fungal pathogens (Forrester and Foster, 2012). Genes coding Fhb-like proteins have also been identified in fungal phytopathogens, including *B. cinerea*, *Cladosporium fulvum*, *F. oxysporum, Gibberella zeae*, and *N. crassa* (Boccara et al., 2005); however, the *in vivo* ability to detoxify NO via NO-dioxygenase activity has only been confirmed for *B. cinerea*. Unlike human pathogens, deletion of *Bcfhg1* did not affect pathogenicity of *B. cinerea* in relation to three different host species such as *S. lycopersicum*, *A. thaliana*, and *P. vulgaris* (Turrion-Gomez et al., 2010).

Another system contributing to nitrosative stress resistance employs *S*-nitrosoglutathione reductase (GSNOR). This GSH-dependent bi-functional enzyme is able to reduce GSNO to form GSSG plus NH_3_, as well as detoxify formaldehyde (Tillmann et al., 2015). In plants, GSNOR modulates the extent of cellular *S*-nitrosothiol (SNO) formation following nitrosative stress and is required for disease resistance (Feechan et al., 2005; Yu et al., 2012). As it was mentioned before, in *M. oryze* a *MoSFA1* coding *S*-(hydroxymethyl)glutathione dehydrogenase belonging to a class III alcohol dehydrogenase was proven to be involved in NO metabolism *via* GSNO reduction (Zhang et al., 2015). As evidenced by Fernandez and Wilson (2014), GSH-recycling in the GSH-dependent antioxidant system is critical in the colonization phase of host cells by *M*. *oryzae*. Moreover, loss of *MoSFA1* increases the level of SNOs creating indirectly NO over-accumulation and significantly attenuated the virulence on plant hosts (Zhang et al., 2015). It worth pointing that both enzymes counteracting nitrosative stress, i.e., Fhb and GSNOR, promoted virulence of the human fungal pathogen *Cryptococcus neoformans* (de Jesús-BerraaaIos et al., 2003).

An unknown strategy to remove NO or suppress its excessive accumulation has been suggested for *G. orontii*. In *Arabidopsis* leaves infected with the host-adapted powdery mildew a localized and high peak of NO formation coincided in time with appressorium formation by *G. orontii* primary hyphae. Since the NO level rapidly declined to the background level after the initial burst, the biotrophic pathogen might actively modulate its amount by degradation/decomposition mechanisms and in consequence resume growth and colonize the whole leaf (Schlicht and Kombrink, 2013). In *Candida albicans*, a human fungal pathogen, RNS exerts fungistatic effects probably by causing damages through *S*-nitrosylation of proteins and glutathione. In response to this stress, a set of genes, including RNS detoxifying enzyme, is induced. Following RNS detoxification, *C. albicans* restored redox homeostatis and the resulted *S*-nitrosylated adducts could be repaired *via* denitrosylation, allowing the pathogen to resume growth (Brown et al., 2014).

It is worth pointing out that screening of a genomic DNA library makes it possible to identify a *ntpA* gene that conferred growth tolerance upon *A. nidulans* exposure to exogenous NO (Zhou et al., 2013). The *ntpA* disruption increased amounts of cellular SNO and provoked NO susceptibility. The gene coding a cysteine-rich 23-amino-acid peptide that reacts with NO and GSNO to generate an *S*-nitrosated peptide called inducible nitrosothionein (iNT). The NO scavenging role of iNT seems to be mediated by thioredoxin-dependent catalysis. Importantly, the authors highlighted that the ubiquitous distribution of iNT-like polypeptides constitutes a potent NO-detoxifying mechanism that is conserved among various organisms (Zhou et al., 2013).

## Conclusion

Increasing knowledge from diverse systems indicates that NO plays a pivotal role in the immune response of plants attacked by pathogens with different lifestyle. However, despite extensive research on NO synthesis and signaling processes in plants interacting with biotic stressors, our understanding of NO in phytopathogens is very limited. What is more, a validity of the reports on the biological action of NO in pathogenic microorganisms should undergo some criticism due to the methods used for NO detection. For example, the specificity of widely used DAF-based dyes is questionable since under biological conditions the fluorescent triazole product can be formed from either nitrosative or oxidative chemistry (Bryan and Grisham, 2007). Also the specificity confirmation of NO-dependent fluorescence using the NO scavenger 2-(4-carboxyphenyl)-4,4,5,5-tetramethylimidazoline-l-oxyl-3-oxide (cPTIO) need to be used with caution because there is evidence that it can increase fluorescence (Arita et al., 2007). Finally, the use of NOS inhibitors in looking for NOS activity in fungal and oomycete pathogens does not provide an unequivocal confirmation for the enzyme presence, since these chemicals can inhibit other biosynthetic pathways as well (Planchet and Kaiser, 2006).

**FIGURE 1:**
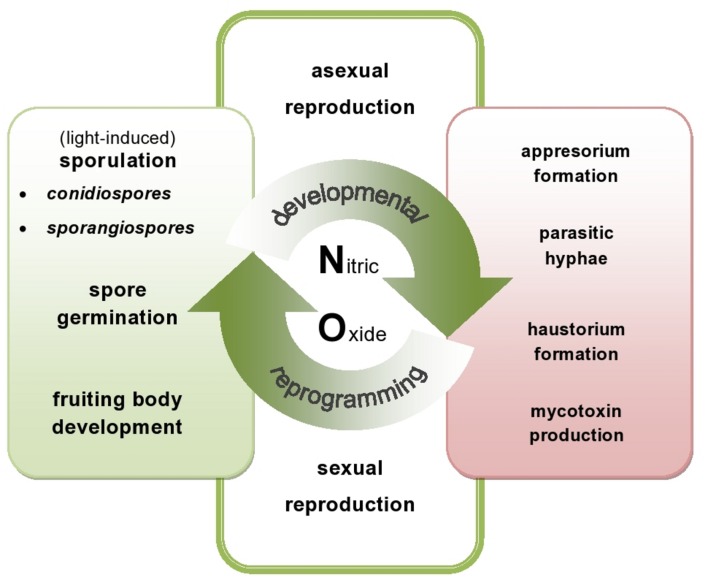
**Nitric oxide (NO)-mediated events in plant pathogenic fungi and oomycete**. NO might be directly or indirectly involved in the switch between developmental phases of the phytopathogens. On the one hand, endogenously produced NO participates in various developmental processes including sporulation, germination and fruiting bodies formation (green area – NO in developmental events). On the other hand, NO may be responsible for production of toxins and differentiation of various infection structures of the pathogens (red area – NO in offensive strategy).

Fungi and oomycete pathogens have active sources of NO and pathways of its detoxication, which defend them against NO-induced damages and ensure the vital level required for the signaling function both in the pathogen physiological state and during host tissue colonization (**Figure 1**). Although pathogens with different lifestyles vary in their sensitivity to host-generated RNS, a flexible NO metabolism could constitute a good strategy to subdue the host plant.

Recognition of pathogen-derived NO during the battle between both adversaries will help us to resolve one of the central questions in plant pathology, namely what makes a pathogen successful and what makes a plant become a vulnerable host. To this end there are many important issues which need to be addressed. Firstly, research on additional genetic resources to unravel NO biosynthesis is needed. Secondly, the identification of cellular targets, degradation pathways and the mechanism/s of signal transduction need to be elucidated within different pathogen structures. Finally, fungal and oomycete pathogens, simultaneously exposed to combinations of different stimuli within the host cells, rather than to RNS alone, could activate specific NO sensing mechanisms relevant to the successful host colonization.

## Author Contributions

MA-J and JF-W provided the idea, wrote the paper. All authors have read and approved the manuscript.

## Conflict of Interest Statement

The authors declare that the research was conducted in the absence of any commercial or financial relationships that could be construed as a potential conflict of interest.
